# Bridging Communication Gaps in Oncology: Insights From a Questionnaire Among Internal Medicine and Intensive Care Physicians

**DOI:** 10.7759/cureus.77670

**Published:** 2025-01-19

**Authors:** Marina Gonçalves, Rita Gouveia, Miguel Barbosa, Sara Meireles

**Affiliations:** 1 Medical Oncology, Centro Hospitalar Universitário de São João, Porto, PRT; 2 Internal Medicine, Centro Hospitalar Universitário de São João, Porto, PRT

**Keywords:** accessibility, communication barriers, intensive care, internal medicine, terminology

## Abstract

Introduction

Oncology has rapidly evolved over the past decades in terms of treatments and outcomes. However, it has also introduced challenges, particularly for non-oncologist physicians who, at some stage of the disease, encounter oncology patients. The increasing complexity of terminology, lack of updated knowledge, or insufficient training, along with other interdisciplinary communication barriers, can lead to less-informed clinical decisions, resulting in suboptimal treatment management.

Objective

This study aims to investigate knowledge and communication limitations with oncology among internal medicine and intensive care physicians to identify educational and operational gaps.

Methods

This was a quantitative study using a questionnaire administered to internal medicine and intensive care specialists and residents (third to fifth year) from three centers. Data was analyzed using SPSS software.

Results

A total of 91 responses were obtained (69 (75.8%) from internal medicine and 22 (24.2%) from intensive care), of which 76.9% (n=70) were specialists. Approximately 92.3% (n=84) reported daily or weekly contact with oncology patients. Fifty-eight percent (n=53) rated their knowledge of oncology terminology as “adequate,” and 93.4% (n=85) disagreed that "first-line palliative treatment" and "first-line metastatic treatment" have the same meaning. The majority (72.5%, n=66) considered the terminology used to be “unclear,” particularly in relation to prognosis. In 72.5% (n=66) of cases, respondents reported that clinical decisions could have been altered if more objective terminology had been used. More than 90% (more than n=82) supported the need for standardized oncology terminology and clinical summaries with prognostic information. Around 93.4% (n=85) stated that continuous education in oncology should be more accessible.

Conclusions

The study revealed significant gaps in communication and knowledge of oncology among non-oncologist physicians, highlighting the need for standardized terminology and greater accessibility to continuous education. These measures could improve oncology care and potentially influence health policies.

## Introduction

Medical oncology is a rapidly evolving field of medicine that has made significant progress in treatments over the past few decades, leading to improved patient outcomes. Scientific and technological advancements have diversified and enhanced cancer treatment options, resulting in markedly better survival rates for various malignancies. However, this progress has also introduced challenges, mainly for non-oncologist physicians who meet cancer patients at different stages of the disease and manage their acute complications, related or not to the cancer itself.

The increasing complexity of oncological terminology and treatment strategies requires up-to-date knowledge to ensure informed and effective clinical decisions. Recent studies suggest that non-oncologist physicians often face difficulties in preventing and managing cancer patients, frequently due to a lack of updated knowledge or specific training. Notably, research has shown that non-oncology doctors' understanding of oncology is often not current, which can lead to risks in communication with patients [1].

Furthermore, the prognosis of patients may be impacted by unclear information regarding the continuity of oncological care across different points in the healthcare network. The delays in starting oncological treatment often arise from communication and coordination issues among medical teams, and it could be associated with reduced survival outcomes for certain types of cancer [2].

Effective communication between physicians and patients is crucial, especially when discussing oncological diagnoses and prognoses. The Medical Ethics Code emphasizes the value of informing the patient about the diagnosis, prognosis, risks, and treatment aims, except when such communication could be harmful; in these cases, the information should be provided to the legal representative.

Therefore, non-oncologist physicians must receive adequate training and regular updates on oncological advances and prognosis. This might enable better care integration, more informed clinical decisions, and improved patient outcomes.

## Materials and methods

The study was conducted through a questionnaire entitled “Questionnaire to Assess Communication Barriers Between Non-Oncologist Physicians and Medical Oncology,” which was applied after approval by the Ethics Committee of the Local Health Unit of São João. The target population included medical specialists or trainees in the third to fifth year of specialty training in internal medicine and intensive care medicine. The questionnaire encompassed physicians from the local health units of São João, Médio Ave, and Matosinhos. Physicians from internal medicine were included regardless of whether they worked in inpatient wards, emergency services, or intermediate care units.

The inclusion of internal medicine and intensive care specialties in the study was based on the fact that oncology patients are typically managed within the realm of medical specialties. These two disciplines were selected due to their significant role in influencing therapeutic decisions during acute complications that may arise in cancer patients. Such acute events can substantially impact patient prognoses, and the involvement of these specialties ensures a comprehensive approach to managing these critical situations.

The questionnaire included five sections with 29 questions, and it was available in both online and physical formats. After signing the informed consent form, the participants completed the following sections: general information, knowledge of oncology terminology, advances in oncology and their impact on decision-making, communication and collaboration with medical oncology, and suggestions and improvements.

Before submission to the Ethics Committee, the questionnaire was reviewed by a panel of 10 medical oncology specialists, two internal medicine specialists, and one intensive care medicine specialist. The feedback from the reviewers, particularly from internal medicine and intensive care specialists, was instrumental in refining the questions to address these specialties' main limitations in their interactions with medical oncology. This optimization aimed to enhance the questionnaire's ability to evaluate communication barriers and obtain meaningful results. The reviewers did not participate as respondents to the questionnaire.

A descriptive analysis of the collected data was performed using IBM SPSS Statistics for Windows, Version 28 (Released 2021; IBM Corp., Armonk, New York, United States). The exclusion criterion was the failure to obtain a response to the informed consent form.

## Results

General information

The study included 91 participants, comprising 69 (75.8%) internal medicine physicians and 22 (24.2%) intensive care physicians. Most participants in both specialties were experienced specialists. The demographic and professional data are summarized in Table 1.

**Table 1:** Demographic and professional characterization of the sample

	Internal medicine physicians (n=69)	Intensive care medicine physicians (n=22)
Gender - n (%)		
Male	28 (40.6%)	13 (59.1%)
Female	41 (59.4%)	9 (40.9%)
Specialist - n (%)	52 (75.4%)	18 (81.8%)
Years of specialization - n (%)		
0-5 years	21 (30.4%)	6 (27.3%)
6-10 years	11 (15.9%)	4 (18.2%)
>10 years	20 (29.0%)	8 (36.3%)
In training - n (%)	17 (24.6%)	4 (18.2%)
Main area of practice - n (%)		
Ward	54 (78.3%)	-
Intermediate care unit (ICU)	11 (15.9%)	-
Exclusively emergency care	4 (5.8%)	-
Frequency of contact with oncology patients - n (%)		
Daily	15 (21.7%)	7 (31.8%)
Weekly	49 (71.0%)	13 (59.1%)
Monthly	5 (7.2%)	2 (9.1%)
The main reason for contacting the oncology patient - n (%)		
Diagnosis and staging	23 (33.3%)	2 (9.1%)
Acute complication to oncological treatment	18 (26.1%)	11 (50%)
Acute complication during the progression phase of oncological disease	14 (20.3%)	3 (13.6%)
Acute complication unrelated to the oncological disease	14 (20.3%)	6 (27.3)

Knowledge of oncology terminology

The majority of participants assess their knowledge of oncology terminology as adequate (Table 2). When asked about which terms are considered part of a patient's curative treatment, most of them identified key terms such as "neoadjuvant," "adjuvant," and "primary" (Table 3). Less common terms like "conversive" were less frequently recognized (Table 3).

**Table 2:** Evaluation of oncological knowledge, accessibility, and terminology clarity

Question	Excellent and good/very accessible/very clear - % (n)	Adequate/accessible/clear - % (n)	Insufficient and poor/not very accessible/not very clear - % (n)	Not accessible at all/confusing - % (n)
How do you assess your knowledge of current oncological terminology?	16.5 (15)	58.2 (53)	25.3 (23)	—
How effective do you consider your hospital’s oncology service in terms of accessibility?	17.6 (16)	50.5 (46)	30.8 (28)	1.1 (1)
What is your opinion about the clarity of the oncological terminology used in reports/medical records by medical oncology?	2.2 (2)	35.2 (32)	50.5 (46)	12.1 (11)

**Table 3:** Terms considered as part of the course of a patient undergoing curative treatment

Term	% (n)
Neoadjuvant	80.2 (73)
Primary	73.6 (67)
Adjuvant	82.4 (75)
Conversive	25.3 (23)
First-line palliative	4.4 (4)
First-line metastatic disease	25.3 (23)

Regarding the interpretation of oncological terms, most participants (93.4%, n=85) disagreed that "first-line palliative treatment" and "first-line metastatic treatment" have the same meaning. Additionally, the large majority (80.2%, n=73) agreed that the term "prognosis-modifying treatment" provides a clearer clinical message compared to "palliative treatment."

Advances in oncology and impact on decision-making

As reported by most of the participants (81.3%, n=74), the primary goal of treating metastatic oncological disease is to prolong the patient's life by controlling tumor growth and maintaining quality of life. A minority (12.1%, n=11) considered symptom control, without survival impact or curative intent, as the primary aim.

Regarding the palliative systemic treatment, all respondents disagreed that only one effective line of treatment is typically available in this context. However, 22% (n=20) of them believed that patients undergoing palliative systemic therapy have a survival time of less than 12 months.

Most participants indicated that concurrent follow-up in outpatient palliative care clinics frequently influences their treatment perspectives (40.7%, n=37) (Table 4).

**Table 4:** Impact of concurrent outpatient palliative care on treatment perspective

Impact of palliative care follow-up on therapeutic decision-making for metastatic patients	% (n)
Frequently	40.7 (37)
Occasionally	35.2 (32)
Rarely	19.8 (18)
Never	4.4 (4)

Most of the respondents (81.3%, n=74) felt they were not up-to-date with advances in oncology that positively impact survival. Additionally, 90.1% (n=82) agreed that metastatic cancer patients undergoing palliative treatment are generally not considered candidates for care escalation due to poor prognosis.

The key factors that influence decisions to escalate care to level II/III include metastatic disease (50.5%, n=46, with significant impact) and third-line treatment or beyond (75.8%, n=69, with significant impact). Conversely, patient age and non-oncological complications had little impact (Table 5).

**Table 5:** Factors affecting the decision to escalate care to level II/III

% (n)	No impact	Little impact	Moderate impact	Significant impact
Metastatic disease	0	11 (10)	38.5 (35)	50.5 (46)
Locally advanced disease	9.9 (9)	42.9 (39)	39.6 (36)	7.7 (7)
Stable metastatic oncological disease	13.2 (12)	42.9 (39)	31.9 (29)	12.1 (11)
Patient in the second line of treatment for metastatic disease	4.4 (4)	13.2 (12)	48.4 (44)	34.1 (31)
Patient in the third line of treatment for metastatic disease	0	5.5 (5)	18.7 (17)	75.8 (69)
Median life expectancy > 6 months	6.6 (6)	30.8 (28)	29.7 (27)	33 (30)
Median life expectancy > 12 months	30.8 (28)	35.2 (32)	17.6 (16)	16.5 (15)
Intercurrence related to disease progression	13.2 (12)	22 (20)	45.1 (41)	19.8 (18)
Intercurrence not related to the oncological disease	40.7 (37)	42.9 (39)	11 (10)	5.5 (5)
Age > 60 years	53.8 (49)	36.3 (33)	5.5 (5)	4.4 (4)

Communication and collaboration with medical oncology

The majority of respondents rated the accessibility of the oncology service as adequate or very accessible, while a minority considered it insufficient (Table 2).

Most of the participants found the oncological terminology used in medical reports unclear, with only a small portion describing it as very clear (Table 2). Additionally, several respondents reported difficulties in understanding specific technical terms, treatment protocols, and disease staging (Table 6).

**Table 6:** Main challenges to understanding oncological nomenclature

Question	Specific technical terms - % (n)	Staging of the disease - % (n)	Treatment protocols - % (n)	The current state of the disease - % (n)	Prognosis - % (n)
What are the main challenges you face regarding oncological nomenclature?	47.3 (43)	30.8 (28)	73.6 (67)	52.7 (48)	72.5 (66)

A significant majority of respondents (69.2%, n=63) indicated that a limitation of communication with medical oncology was the insufficient knowledge of oncologists regarding the outcomes of patients after ICU or intermediate care unit discharge and their subsequent impact on oncological treatments. Furthermore, 72.5% (n=66) of respondents believed that in certain situations, they could have made a different decision if the clinical information had been more objective.

Suggestions and improvements

Nearly all respondents (95.6%, n=87) agreed that standardizing and simplifying oncological terminology would improve interdisciplinary communication. All participants supported that the inclusion of a summary of the patient’s clinical situation (covering the current state of the disease, treatment, and survival prognosis) in the last consultation report could be a beneficial tool for emergency settings. Additionally, 93.4% (n=85) of respondents indicated that continuous education in oncology should be more accessible within their area of expertise.

## Discussion

The results of this study highlight significant barriers in communication between non-oncologist physicians and medical oncologists, with potential impacts on the quality of care provided to oncology patients.

The primary limitation identified by participants was insufficient knowledge of oncological terminology and the most recent therapeutic advances. These factors contribute to difficulties in interpreting medical reports, making clinical decisions, and fostering interdisciplinary communication [3]. One key example is the term "palliative treatment," which is often misunderstood as synonymous with low survival rates, leading to misjudgments about the goals of care and potential therapeutic options. This is highlighted by the fact that 93.4% (n=85) of participants disagreed that "first-line palliative treatment" and "first-line metastatic treatment" have the same meaning.

The majority of interviewers have the perception that the terminology used by oncologists is "not very clear" or "confusing," reflecting a failure to effectively convey essential information for integrated patient care. Recent studies corroborate this observation, reflecting that the standardization of medical language could facilitate collaboration and reduce communication failures [1,4]. The simplification and regular training were unanimously recognized by the participants as being effective measures to address these limitations.

A clear example of this difficulty lies in the interpretation of the terms "palliative" and "metastatic." Most of the participants acknowledged that these terms have distinct meanings and that their correct use is essential to communicate the therapeutic goal and disease status. However, their responses indicated that the distinction between "first-line palliative treatment" and "first-line metastatic disease treatment" is not always clear to all involved professionals. The most important change, considering this finding, is the standardization of the use of these two terms. This confusion can lead to misunderstandings regarding treatment goals, potentially resulting in delays in initiating appropriate therapies or the implementation of treatments that are not aligned with the patient's actual clinical needs [5,6].

Another critical point was the lack of updated knowledge in oncology. Most of the non-oncologist physicians reported not being updated in areas where significant advances have been made in improving survival rates. This finding reinforces the need for continuous educational initiatives, such as specific training courses and updated programs, which could be implemented as part of professional development for physicians in clinical areas related to oncology [7,8].

In addition, the lack of objective information in intensive care discharge reports was identified as a critical obstacle. More than two-thirds of participants indicated that greater clarity and objectivity could have influenced their previous clinical decisions. This finding highlights the importance of including a standardized summary of the patient's condition in all reports, detailing the current state of the disease, the treatment line, the intent of treatment (curative or metastatic), and the estimated prognosis (greater or less than six months) - a measure widely recognized as beneficial in similar studies [7,8].

The frequency of contact with oncological patients also influenced participants' responses. While intensive care physicians reported greater involvement in acute complications related to disease progression, internal medicine physicians were more frequently involved in diagnosis and staging. These differences reflect the complexity of oncology care, suggesting the need for a collaborative and multidisciplinary approach to optimize outcomes [9].

Finally, it is important to recognize that, although this study included a representative sample of physicians from the specialties of internal medicine and intensive care, our limitations include the sample size and the fact that it was conducted in three local health units. With broader studies, we could better understand where the core issues truly lie, beyond terminology, and implement more cross-cutting changes to address them effectively.

## Conclusions

This study highlights significant barriers in communication between non-oncologist physicians and medical oncology. This translates into limited knowledge of oncological terminology, difficult-to-interpret medical reports, and insufficient familiarity with recent advances in oncology. Misunderstood terms like "palliative" and those linked to curative intent, such as "conversive," "neoadjuvant," and "primary," contribute to these challenges. Additionally, there is limited awareness of the benefits of concurrent palliative care in improving patient outcomes. These gaps impact effective decision-making and interdisciplinary collaboration, ultimately affecting patient outcomes. The results underscore the importance of standardizing and simplifying oncological terminology to enhance clarity and the integration of clinical summaries into medical reports, including treatment details and prognostic information, for better emergency care management.

Furthermore, the findings reveal a strong demand for more accessible and tailored continuous oncology education for non-oncologist physicians to address these challenges. Examples include the development of hospital-based interdisciplinary action protocols for managing complications in oncology patients, case discussions of shared clinical scenarios, internal training programs, and the integration of oncology rotations within specialty training curricula. Implementing these strategies can bridge communication gaps, promote cohesive care, and improve the quality of oncology services. Future research should explore these interventions in larger, diverse cohorts to confirm their effectiveness and scalability.

## Appendices

Questionnaire to assess communication barriers between non-oncologist Physicians and Medical Oncology

 *Section 1: General Information*

1. What is your specialty?

 - Internal Medicine

 - Intensive Care Medicine (Specialist or Study Cycle in Intensive Care Medicine)

 - Specialized Training Intern in Internal Medicine

 - Specialized Training Intern in Intensive Care Medicine

2. If you are a Specialized Training Intern, what year of training are you in?

 - 3º

 - 4º

- 5º

3. If you are a Specialist, how many years have you been practicing?

 - 0-5 years

 - 6-10 years

 - 11-15 years

 - More than 15 years

4. If you are an Internal Medicine Doctor, where do you primarily practice your duties?

 - Internal Medicine Ward

 - Intermediate Care Unit

 - Stroke Unit

 - Emergency Department (exclusively)

5. In which hospital do you practice?

 - Local Health Unit of Matosinhos

 - Local Health Unit of Médio Ave

 - Local Health Unit of São João

6. How frequently do you treat oncological patients in your clinical practice?

 - Daily

 - Weekly

 - Monthly

 - Rarely

7. In which stage of the disease does contact with the oncological patient typically occur? Rank in ascending order of frequency (with 1 being the least frequent and 4 being the most frequent)

 - Diagnosis/Staging

 - Acute treatment-related complications (toxicity/infections)

 - Complications during disease progression

 - Complications unrelated to the oncological disease

Section 2. Knowledge of Oncology Terminology

1. How do you assess your knowledge of current oncological terminology?

 - Excellent

 - Good

 - Adequate

 - Insufficient

 - Poor

2. Which of the following terms do you understand as part of the treatment pathway for an oncological patient with the intent to cure?

 - Adjuvant treatment

 - Primary treatment

 - Neoadjuvant treatment

 - First-line palliative treatment

 - Conversion intent treatment

 - First-line treatment for metastatic disease

3. In your understanding, do the terms "first-line palliative treatment" versus "first-line metastatic treatment" have the same connotation/meaning?

 - Yes

 - No

4. Considering the evolution in oncology disease outcomes, does the expression "prognostic-modifying treatment" versus "palliative treatment" reflect any improvement in the clarity of clinical information?

 - Yes

 - No
 

Section 3: Advances in Oncology and Impact on Decision-Making

1. What do you consider to be the main goal of treating metastatic oncological disease?

 - Cure the disease.

 - Prolong the patient's life by controlling tumor growth and maintaining quality of life.

 - Provide symptom control for the oncological disease, but with no impact on survival.

 - Avoid any medical interventions.

2. In most neoplasms, patients undergoing systemic treatment with a palliative intent only have one effective treatment line available. Do you consider this statement:

 - True

 - False

3. Oncological patients undergoing systemic treatment with a palliative intent typically have an overall survival time (from the diagnosis of the oncological disease to death) of less than 12 months. Do you consider this statement:

 - True

 - False

4. If an oncological patient with metastatic disease undergoing active treatment is also being followed up in a Palliative Care Outpatient Clinic, do you believe this has an impact on your perspective of the patient's treatment?

 - Frequently

 - Occasionally

 - Rarely

 - Never

5. Do you consider yourself relatively up-to-date in the areas of oncology where treatments have had a positive impact on survival?

 - Yes

 - No

6. For you, in oncology patients, which of the following criteria have negatively influenced your decision to escalate care to level II/III?

Please rate each one individually from 1 to 4 by marking an “x” (where 1 = no impact, 2 = low impact, 3 = moderate impact, and 4 = high impact).

**Table 7:** Question

	1	2	3	4
Metastatic disease				
Locally advanced disease				
Stable metastatic oncological disease				
Patient in the second line of treatment for metastatic disease				
Patient in the third line of treatment for metastatic disease				
Median life expectancy > 6 months				
Median life expectancy > 12 months				
Intercurrence related to disease progression				
Intercurrence not related to the oncological disease				
Age > 60 years				

7. Do you think the general opinion of the medical community is that a metastatic cancer patient undergoing palliative treatment is not indicated for escalation of care, due to the poor prognosis associated?

 - Yes

 - No

Section 4: Communication and Collaboration With Medical Oncology

1. Do you consider the accessibility to the Oncology Service at your hospital effective?

 - Very accessible

 - Accessible

 - Not very accessible

 - Not accessible at all

2. Do you consider a limitation to communication with Medical Oncology the limited knowledge of oncologists about the outcomes of patients after ICU/Intermediate Care Unit discharge and their impact on subsequent oncological treatments?

 - Yes

 - No

3. What is your opinion on the clarity of the oncological terminology used in reports/medical records by Medical Oncology?

 - Very clear

 - Clear

 - Not very clear

 - Confusing

4. How often do you seek to contact Medical Oncology for clarifications?

 - Frequently

 - Occasionally

 - Rarely

 - Never

5. What are the main difficulties you encounter regarding oncological nomenclature? (Mark all that apply)

 - Specific technical terms

 - Staging of the disease

 - Treatment protocols

 - Current state of the disease

 - Prognosis

6. Do you think that in any situation, you could have made a different decision if the clinical information had been more objective?

 - Yes

 - No

  6.1. If you answered "yes," how often does this tend to happen?

    - Frequently

    - Occasionally

    - Rarely

Section 5: Suggestions and Improvements

1. Do you think that the standardization and simplification of oncological terminology would benefit interdisciplinary communication?

 - Yes

 - No

2. Do you believe that having a summary of the patient's clinical situation (current state of the disease, treatment, and prognosis of survival > or < 6 months) in the report of the last consultation would be beneficial in emergencies? 

 - Yes

 - No

3. What type of resources or tools would help you stay updated on oncological terminology and treatments? Please select all that apply

 - Update courses at your hospital

 - Interdisciplinary training

 - Updated hospital guidelines from the Oncology Service

 - Action Protocols

 - Periodic meetings (monthly, quarterly, or semi-annually)

4. Do you think that continuous education in oncology should be more accessible, considering your area of expertise?

 - Yes

 - No
